# Annotations

**Published:** 1906-01-20

**Authors:** 


### ANNOTATIONS.

### Sir William Collins, M.P.

A SATISFACTORY feature of the General Election which is still in full swing is the return for West St. Pancras of Sir William Collins. In referring to his candidature last month we strongly urged his claims, not, of course, because of his political convictions, which do not affect this journal, but because of the public services he has rendered, and because of his recognised position in the medical world. The new House of Commons, like its predecessors, will be all the better for the presence, at Westminster, of men who have displayed exceptional ability in the pursuit of their own calling or profession, and exceptional administrative skill in the management of the institution with which they have been prominently associated. Sir William Collins, as we pointed out on a former occasion, answers in every respect to this description. It is because we are satisfied that he will be no mere party hack, but will bring to bear upon his new duties as a legislator the sound judgment, the keen intelligence, and the ripe knowledge, which he has displayed in other scenes of activity, that we unfeignedly rejoice at his success. To these may be added the fact that he possesses an extremely charming personality, is a resourceful and eloquent speaker who is likely to make his mark as a debater, and whatever his views, has the courage to give frank expression to them. It is probable that several questions in which medical men are greatly interested will come before the Legislature at an early date, and Sir William Collins will be one of the far too few representatives of the profession who will be able to treat them from the standpoint of an expert. There is no doubt that in the past Bills concerning the public health have failed to become law mainly owing to the absence of men who combine technical experience with the energy in action which is so essential.

### The Diminishing Birth-rate.

THIS phenomenon continues to exercise the minds of all who can look beyond the narrow circle of the immediate national future, and not without cause. To the speculatively inclined the contemplation of the falling figures of our birth-rate may well conjure up a vision reminiscent of barbaric expansions in historic times, of races prolific, hardy, and of few wants, turning their steps towards the rich fields of enervated civilisation, and overrunning them by sheer weight of numbers. To the less imaginative citizen it may possibly occur that there is something to be said on the side of a reduction in the teeming population of our cities; advantage, that is, to the individual, though, possibly, at the cost of the race. Sociologists, again, have to reckon with the circumstance that this reduction in births is not evenly distributed among the several broad ranks into which our society is divided, but is principally evident among those of higher education and greater general culture, a fact which further complicates the social aspects of the problem. The reason of this disproportion is not far to seek, for to the cultured classes children are an earnest of great expenditure to be demanded by their education, while to the labourer children are an assurance against destitution in old age. Moreover, with the progressive spread of knowledge concerning the principles of heredity, it is probable that conscientious considerations to which the labourer is a total stranger assist in modifying the prolificity of the cultured, although there must doubtless be considered, in addition to these factors, a disinclination to make the sacrifices of personal freedom and indulgence which are inseparable from, and important educational elements of, the married state. This is a matter in which the interest of the individual clashes with the interest of the race; in which, for once, prudence and patriotism are antipathetic. It is hard to blame a man for declining to bring into the world children for whom he is unable to make suitable provision, and yet this forethought is probably the most weighty of the causes operating to produce the existing state of affairs.

### How to Prevent Boys from Smoking.

IT may be some years before Parliament can be induced to pass a Bill to render it a penal offence to sell tobacco in any form to individuals of tender age. There is a curious indisposition on the part of the Legislature to interfere with the liberty of the subject, even if he does not possess a vote and is not capable of taking proper care of himself. But we freely concede that there is a better way of dealing with the ever-increasing evil of smoking by growing lads than by legislation. It has been shown by a tobacconist in Bromley, who has just issued a notice that he will no longer supply cigarettes and tobacco to boys under the age of sixteen. This excellent tradesman states that during the Christmas season he made up his mind to take action. Modestly admitting that he does not know whether it is true that smoking stunts growth, he says he is sure that it must be injurious to boys physically and mentally, and he is also afraid, from observation and experience, that the money devoted to the purchase of cigarettes is not always honestly obtained. He adds that though he has fixed sixteen as the limit under which he will not serve lads, he does not suggest that that is the proper age for them to start smoking. The limit, if legislation were in question, would be too low; but the Kentish tobacconist has done well not to err on the side of severity. Refusing to echo the selfish cry of the ages, "Am I my brother's keeper?" he has addressed himself to the nobler task, at the probable cost of loss of income and popularity, of attempting, by personal example and self-sacrifice, to do the best he can to arrest a habit which experts know to be frequently attended by disastrous results. If every tobacconist would have the courage and the patriotism to follow it, a potent cause of physical and mental national deterioration would be practically removed.

